# Clinical utility of 24-h rapid trio-exome sequencing for critically ill infants

**DOI:** 10.1038/s41525-020-0129-0

**Published:** 2020-05-05

**Authors:** Huijun Wang, Yanyan Qian, Yulan Lu, Qian Qin, Guoping Lu, Guoqiang Cheng, Ping Zhang, Lin Yang, Bingbing Wu, Wenhao Zhou

**Affiliations:** 1 Center for Molecular Medicine, Children’s Hospital of Fudan University, 201102 Shanghai, China; 2 Pediatric intensive care unit, Children’s Hospital of Fudan University, 201102 Shanghai, China; 3 Department of Neonates, Key Laboratory of Neonatal Diseases, Ministry of Health, Children’s Hospital of Fudan University, 201102 Shanghai, China

**Keywords:** Molecular medicine, Genetic testing

## Abstract

Genetic diseases are a leading cause of death in infants in the intensive care setting; therefore, rapid and accurate genetic diagnosis is desired. To validate 24-h trio-exome sequencing (TES), samples from probands and their parents were processed by the AmpliSeq /Ion S5XL platform in a hospital clinical laboratory. Infants from the intensive care unit (ICU) suspected of having a genetic disease were enrolled. Regular and 24-h TES using the Agilent SureSelect capture kit/Illumina platform were performed on all samples in parallel. Of 33 enrolled infants, 23 received positive results with rapid TES, and an additional two diagnoses were achieved with regular TES. Among the 23 diagnosed patients, 10 experienced changes in medical management, such as hematopoietic stem cell transplant. Ten diagnosed cases were discharged prior to receiving the regular TES results; six received timely symptom control, and four withdrew medical support. Rapid TES enabled faster time to diagnosis, which resulted in an overall decrease in length of hospital stay. The 24-h TES can serve as a rapid response tool for patients with suspected monogenic disorders and can guide clinical decision-making in urgent cases.

## Introduction

Genetic disease diagnosis in infants can be extremely challenging to diagnose because the symptoms and characteristics may not appear early in life and may progress rapidly^1–3^. It is critical to diagnose these children as soon as possible to enable timely interventions, thus reducing mortality and unessential intensive care, as well as minimizing the anxiety of patient families. Patients without a diagnosis often embark on a diagnostic odyssey, including multiple specialist consultations, invasive investigations, or further laboratory tests, imposing both economic and physical burdens on patients and their families, sometimes even after the patient is deceased^4,5^.

Currently, comprehensive clinical panel testing or exome sequencing requires weeks to obtain genetic test results^6,7^. It is difficult to break the 1-week turn-around-time (TAT) barrier because conventional high throughput sequencing platforms run cases by batch, reducing flexibility for individual cases. In principle, it has been shown that genome sequencing as a first-tier genetic test can provide a higher diagnostic yield than conventional genetic testing in a clinically heterogeneous cohort^8^. Furthermore, rapid genome sequencing can decrease infant morbidity and hospitalization costs in acutely ill inpatient infants^4,9,10^. However, current rapid trio-genome sequencing is cost-prohibitive for most patients in developing countries. Moreover, data analysis of genomes has not fully matured due to its high complexity. Therefore, because rapid trio-exome sequencing can, by comparison, produce results in a short amount of time and is less expensive, it is the ideal testing strategy for critically ill infants in the pediatrics/neonate intensive care unit (PICU/NICU).

Here we present a rapid 24-h trio-exome sequencing (TES) pipeline that permits early genetic diagnosis with a TAT of approximately 24 h, which is comparable to the record of rapid genome sequencing^9,11^ but with only a small fraction of its cost. Among a group of critically ill infants who were highly likely to have diseases of genetic etiology, we successfully identified molecular diagnoses in 23 of 33 cases. Furthermore, treatment plans were modified in 10/23 of the diagnosed patients, and the overall hospitalization time decreased in four NICU/PICU patients. Overall, this study showed that clinical trio-exome can be achieved with a 24 h TAT and at a reasonable cost and may enhance the clinical utility of genomic sequencing for critically ill patients.

## Results

### Clinical characteristics

Twenty-two males and 11 females, ranging in age from 2 to 210 days old, with a mean of 55.97 days and a median of 51 days were enrolled in this study. Ten of the participants were neonates from the NICU, and 23 were from the PICU. There were 14 (43%) patients with metabolic-related diseases, 8 (24%) of whom had neuromuscular disorders, 8 (24%) of whom had immunodeficiency diseases, and 3 (9%) of whom had dysmorphic conditions with multiple congenital anomalies. Patients stayed in the ICU from 1 to 72 days, with an average of 21.8 days and a median of 12 days; only 10 (30%) stayed less than one week (Table 1; Supplementary Table 1–3).

**Table 1:** Demographic and clinical characteristics of the 33 critically ill infants.

Characteristic	Total (*n* = 33)	Diagnosed by rapid TES (*n* = 23)	Undiagnosed by rapid TES^a^ (*n* = 10)
Gender			
Male	22 (66.7%)	15 (65.2%)	7 (70%)
Female	11 (33.3%)	8 (34.8%)	3 (30%)
Age (days)			
≤28	10 (30.3%)	7 (30.4%)	3 (30%)
>28	23 (69.7%)	16 (69.6%)	7 (70%)
Cases diagnosed in the category			
Metabolic-related diseases	14 (42.5%)	11 (78.57%%)	
Neuromuscular disorders	8 (24.2%)	4 (50%)	
Immunodeficiency diseases	8 (24.2%)	5 (62.5%)	
Dysmorphic with multiple congenital anomalies	3 (9.1%)	3 (100%%)	
Average length of ICU stay (days)	21.8	19.4	27.1
Median length of ICU stay (days)	12	8	28.5
Influence of genetic diagnosis on clinical management and outcomes			
Specific treatment	10 (30.3%)	10^b^ (43.5%)	0
With palliative therapy	9 (27.3%)	3 (13 %)	6 (60%)
Withdraw medical support	8 (24.2%)	6 (26.1%)	2 (20%)
Decease in ICU	6 (18.2%)	4 (17.4%)	2 (20%)
The family has subsequent pregnancy plans	2	2	0

^a^Two other cases were diagnosed by regular TES.

^b^One patient (case 32 failed HSCT and died).

### Timeline of the rapid TES

**Fig. 1:**
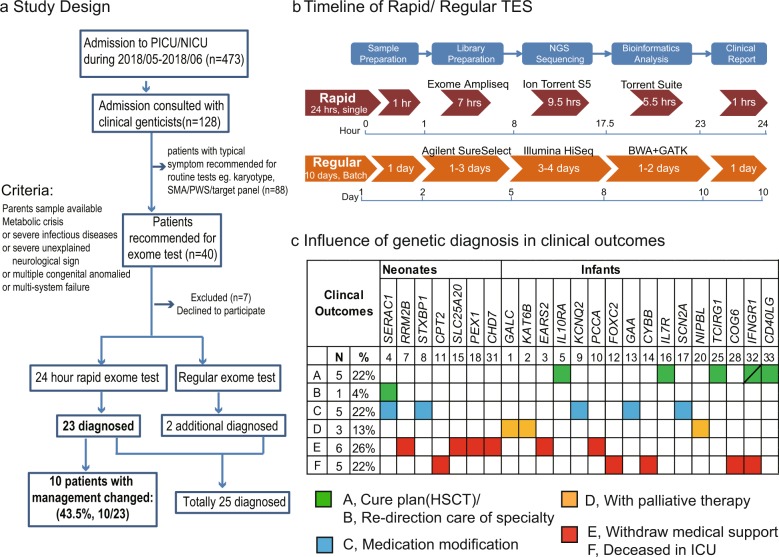
Flow diagram illustrating the frame of rapid TES and clinical outcomes. **a** Study design and flow diagram. **b** Timeline and flow diagram of rapid and regular TES. **c** Diagram of the influence of the genetic diagnosis on the clinical outcomes. The green boxes indicate specific treatment, such as HSCT or redirection (the green box with a diagonal in case 32 indicates that the patient failed HSCT and died); the blue boxes indicate modified medication, orange boxes indicate a change to palliative therapy and the red boxes indicate that the patients withdrew medical support or died in the ICU.

The median TAT for rapid TES was 24 h (22–27 h); it was 10 days (9–12 days) for regular TES (Table 1). The workflow and time requirements for rapid TES were as follows (Fig. 1b): blood samples were sent to the laboratory via the hospital’s internal delivery system, and genomic DNA extraction and DNA quality determination required 1 h. The library preparation and quantification required 7 h. Library enrichment and chip loading took another 7 h. Then, 2.5 h were allotted for sequencing and approximately, 5.5 h for alignment and variant calling in S5 XL servers. The server capacity was expanded to increase the speed of the base alignment and variant calling. VCF files were then loaded on the Fudan Pipeline version 2.0 for automatic analysis and evaluated by senior reviewers. An oral provisional report with candidate pathogenic variants was supplied to the clinician within one hour. Sanger sequencing for patients and parents was performed for positive diagnostic variants. Then, written clinical reports of positive rapid TES cases were issued immediately. For regular TES tests, clinical reports were issued for both positive and negative cases.

### Genetic test results

Rapid TES resulted in the study, 23 of 33 cases with diagnostic findings, including 15 males and eight females, seven (30.4%) were neonates from the NICU, and 16 (69.9%) were from the PICU. Of those receiving a diagnosis, 11 (78.5%) were patients with metabolic-related diseases, four (50%) had neuromuscular disorders, five (62.5%) had immunodeficiency diseases, and three (100%) patients had dysmorphic conditions with multiple congenital anomalies. Detailed information for these patients is provided in Supplementary Table 1. After obtaining the parallel regular TES test results, two additional diagnoses were made in the ten negative cases. The minimal false-negative ratio was 6.06% (2/33). Regular TES identified a homozygous frameshift pathogenic variant of the *MTHFR* gene (NM_005957: c.1267dupG, p.E423Gfs*6) in case 6, which caused homocystinuria due to *MTHFR* deficiency. The rapid TES data provided only three reads in the patient and two reads in the parents at this position. In contrast, in case 19, a hemizygous frameshift pathogenic variant of the *MTM1* gene (NM_000252: c.1446_1447delTG, p.C482*) was identified, causing X-linked myotubular myopathy. The read depths of the pathogenic variant in the *MTM1* gene in rapid TES were lower than 20× in the proband and his parents (11, 8, and 15, respectively, counted in IGV) (Supplementary Table 2).

### Impact of the genetic diagnosis on clinical procedures

The influence of the genetic diagnosis was subdivided into four different outcomes: specific treatment initiation, medication modification, palliative therapy, and deceased. The influence on changed management in ten patients is summarized in Table 2. Five diagnosed patients (case 5: *IL10RA*; case 16: *IL7R*; case 25: *TCIRG1*; case 32: *IFNGR1*; case 33: *CD40LG*) were recommended to undergo hematopoietic stem cell transplant (HSCT). Four patients reached clinical remission, and one patient (case 32) died of multiple organ dysfunction syndromes (MODS) due to the failure of the transplant.

**Table 2:** Changed specific clinical management of ten of 23 genetically diagnosed patients.

ID	Causal gene	Medication change	Surgery change	Avoid examination	Procedure change	Morbidity avoided	Mortality avoided
Case 4	*SERAC1*	Vitamin cocktail and energy mixtures treatment			Monitor blood gas + liver and kidney function + muscle enzyme + hematuria metabolite	Avoided morbidity from symptomatic monitor	Yes
Case 5	*IL10RA*		Avoid overmuch enteroscopy		Changed to HSCT	Avoided severe diarrhea, severe malnutrition	Yes
Case 8	*STXBP1*	Levetiracetam and topiramate was suggested to treat seizure, use one or in combination				Avoid potential neurological damage because of prolonged uncontrolled seizures	Yes
Case 9	*KCNQ2*	Change PB to sodium valproate syrup					Yes
Case 13	*GAA*	Enzyme replacement	Avoid muscle biopsy	Avoid EMG, lab biochemical test and another accessory test			Yes
Case 16	*IL7R*				Early prepared to HSCT	Avoid severe infection	Yes
Case 17	*SCN2A*	Valproate + levetiracetam				Avoid uncontrolled seizures	Yes
Case 25	*TCIRG1*				Changed to HSCT		Yes
Case 32	*IFNGR1*				Changed to HSCT		No
Case 33	*CD40LG*			Avoid overmuch chest radiography, head MRI	Changed to HSCT	Avoid severe infection, pneumonia progress	Yes

*EMG* electromyography, *PB* phenobarbital, *HSCT* hematopoietic stem cell transplantation, *MODS* multiple organ dysfunction syndromes.

Three infants who suffered seizures were found to have de novo pathogenic variants in *SCN2A* (case 17)*, KCNQ2* (case 9) and *STXBP1* (case 8), respectively. These pathogenic variants cause early infantile epileptic encephalopathy. Antiepileptic medicines were adjusted in case 17 with sodium valproate oral solution (Debakin) plus levetiracetam (Caplan), in case 9 with sodium valproate syrup and in case 8 with 10% levetiracetam and topiramate capsules (Topiramate).

Case 4 was genetically diagnosed with *SERAC1* gene mutation, which caused 3-methylglutaconic aciduria with deafness, encephalopathy, and Leigh-like syndrome. The initial symptom was only abnormal coagulation, with no characteristics leading to any clinical diagnosis. After the rapid genetic test, physicians reassessed and confirmed the diagnosis. The patient was then treated with a vitamin cocktail to control the metabolic disorder, and then was arranged to receive a follow-up visit in clinic. Case 13 was diagnosed with glycogen storage disease II, and the patient was discharged on day 8 with suggested treatment of enzyme replacement. With this diagnosis, muscle biopsy, electromyography (EMG), laboratory biochemical tests and other tests were avoided. In addition, case 1 was admitted to the PICU because of infantile muscular hypotonia and seizures and was then discharged after diagnosis of a *GALC* pathogenic variant with follow-up on an out-patient basis (Supplementary Table 1).

The average length of ICU stay in diagnosed patients from rapid TES was 19.4 days, and the median was 8 days (Table 1). Ten cases were discharged before the regular TES results were received, six of whom (cases 1, 8, 9, 13, 17, and 20) received timely symptom control, and four of whom underwent medical support withdrawal (case 3, 10, 15, and 18) (Supplementary Fig. 1, Table 3). These four patients were withdrawn from the ICU within three days after receiving the genetic diagnoses from rapid TES. Compared with the 10 days median TAT of the regular TES test, there were cumulatively at least 31 hospitalization days were saved (8, 7, 7, and 7days, respectively) in these four cases.

### Deceased and undiagnosed cases

Of the diagnosed cases, 11 patients died (Fig. 1c, E, F). In the five patients who died in the ICU, case 11 had lethal neonatal CPT II deficiency; case 12 carried a *FOXC2* mutation, with severe clinical symptoms, and died within 72 h; case 14 had a *CYBB* mutation and died due to BCG vaccine-induced infection before the return of the TES results; case 28, a boy obtained in vitro fertilization, had lethal type III congenital disorder of glycosylation with COG6 mutation, whose parents insisted to keep him in ICU until death at 72 days; and case 32 with compound heterozygous mutation of *IFNGR1* gene, who failed HSCT and died after two months. Six patients were discharged from the hospital and later died (Table 1, Fig. 1c, E). The families of case 3, 10, 15, and 18 withdrew medical support within 72 h after clinical confirmation of the diagnoses of severe metabolic diseases. Case 7 was discharged and died on day 38, case 31 was discharged on day 21 and died shortly after.

Of the eight undiagnosed individuals, two of them died in the ICU on day 1 (case 22) and day 7 (case 23). The families of case 21 and 29 withdrew treatment, and the patients were discharged from the hospital on day 26 and 43 for economic reasons (Supplementary Table 3).

### Patient care and genetic counseling

Special services, such as multidisciplinary team care, palliative care, special diets or medications, imaging studies, surgical procedures, and essential genetic counseling, were provided to the diagnosed families. Two families (case 1 and 18) plan to have another child. Prenatal testing was advised. Eight probands harboring de novo pathogenic variants were also advised to receive counseling before the next pregnancy.

### Phenotype analysis

To evaluate whether special clinical presentations were more likely to be associated with a molecular diagnosis in the ICU, the HPO terms of the diagnosed patients were analyzed. There were 14/24 with neuromuscular problems, 9/17 with metabolic abnormalities, and 8/16 with immune system abnormalities among the diagnosed cases (Supplementary Table 4).

## Discussion

Genetic diseases are the leading causes of death in infants requiring intensive care^12–14^. Here we show the feasibility of rapid clinical exome sequencing to provide genetic diagnoses for a wide range of clinical presentations. Using clinical exome sequencing, some published paper described the genetic diagnoses’ impact on clinical decision making in pediatric patients, and their notable effect on medical management among a group of critically ill infants who were suspected to have genetic disorders^15,16^. It was more efficient than chromosomal microarray detection^17,18^. A systematic literature review (January 2011–August 2017) and meta-analysis in 37 studies, comprising 20,068 children with suspected genetic diseases, showed that the diagnostic rate and clinical utility of WGS/WES were greater than CMA^18^.

The time window for which many life-saving treatments can be most effective for some diseases is very limited. Therefore, developing a rapid diagnostic method for such diseases is an urgent need. The current clinical trio-exome cost is around $1500 with TAT of 2–3 weeks, and the cost of trio-genome sequencing is far higher (around $4500) with similar TAT. Miller et.al.^11^ and Clark et al.^9^ reported a 26-h, and then a 19-h rapid genome sequencing genetic diagnosis system with highly specialized equipment, institute improved analytic tools, and extremely dedicated professionals, which unfortunately cannot be easily replicated in most places. We try to provide a convenient and reproducible rapid trio-WES sequencing for patients stayed in ICU using a personal genome sequencer (Ion Torrent S5 XL) in a laboratory located in the hospital.

In the present study, we developed a 24-h rapid TES for critically ill patients with an acceptable cost of $2200, equivalent to the fee of a 2–3-day stay in the ICU (the daily cost of staying in the ICU was $300–$1000). The main advantage of a 24-h TAT compared to a 2 week TAT is that it enables earlier decision-making and initiation of precise treatments, while also decreasing the length of hospital stay as summarized in Supplementary Fig. 1 and Table 1. Each year, the NICU/PICU at Children’s Hospital of Fudan University admits approximately 6000 patients. The prognosis of certain genetic diseases is favorable if treatment is available. Ten of the 23 (43.5%) experienced a change in medical management. Based on conversations with families, parents of enrolled patients would prefer the rapid TES for quicker results, even with the higher cost compared to a regular exome.

Studies have demonstrated the diagnostic advantage of sequencing trio-families^18–21^. It is difficult to classify the clinical significance of many novel variants without testing the parents. For autosomal dominant inherited disease, the interpretation of de novo variants can be more easily achieved with TES data. In this study, eight patients were diagnosed with de novo pathogenic variants of autosomal dominant genes. Among them, three patients, all of whom showed epileptic seizures caused by different genes, received the most effective medicines after establishing a molecular diagnosis. With data from parents, phases of compound heterozygous variants for autosomal recessive disease genes to avoid calling out variants that are in cis were also immediately available. Furthermore, TES data can also provide paired carrier information for genetic counseling for future family planning^22–24^. Therefore, parental exome data increased the automated analytic speed, by eliminating the time required for Sanger confirmation before provisional reporting. It is possible that the higher cost of diagnostic tests can be offset by reduced length of stay in the NICU/PICU.

In our study, two patients received genetic diagnoses by regular TES in the last 20 days. Case 6 was treated with betaine, folic acid, and cobalamin to control his symptoms. If he was diagnosed by rapid WES, his symptoms maybe have been treated earlier and the length of his ICU stay might have been shorter. For case 19, there was no clinical appropriated treatment for the disease. For these two patients, whose pathogenic variants were missed during initial rapid pipeline analysis, we checked the raw data and found that the variants were in low-depth/low-quality regions, due to the trade-off of high background noise. Similar technical limitations were reported by Lionel et al., who used the Ion Ampliseq Exome Kit for exome amplification and sequencing in the Ion Proton system (Life Technologies)^8^. We plan to improve this limitation by adding more AmpliSeq primers to cover the poor coverage regions, as well as to increase the sequencing depth by using the large capacity chip and vigorous refinement of the bioinformatics algorithms in next step. Another limitation of this study was the CNV calling was not performed due to the limited dataset. However, the CNV analysis results of regular TES data were also negative for the undiagnosed cases.

Overall, this rapid TES method can be performed in the laboratory in the hospital, providing quick and accurate genetic diagnostic technology. In our study, 23/33 critically ill infants in the NICU/PICU received a genetic diagnosis within 22–27 h, that impacted their clinical care including timely amendment of the treatment regimen, initiation of palliative treatment, creation of a long-term treatment program, and genetic counseling.

## Methods

### Clinical sample preparation

This study was performed at the genetic laboratory of Children’s Hospital of Fudan University, Shanghai, China. From May to June 2018, among a total of 473 patients in PICU/NICU, 128 were assessed to likely have genetic diseases by clinical geneticists. Excluding cases with very characteristic features who were recommended for routine tests (*n* = 88), this study enrolled infants with complicated clinical manifestations. The enrollment criteria were as follows: (1) patients with a serious illness that progressed fast but without a definite diagnosis, such as inborn metabolic-related diseases, neuromuscular disorders, immunodeficiency diseases, dysmorphic with multiple congenital anomalies, and previous family history; (2) both parents were available, and routine trio-exome sequencing tests were ordered by clinical geneticists; (3) patient families agreed and signed the informed consents. Forty patients were recommended to receive the exome test, seven of them were excluded because of parents declined to participate. Thirty-three patients met the enrollment criteria (Fig. 1a). These samples were sequenced in parallel using both rapid and regular TES tests. The rapid TES is designed as preclinical research with no additional cost to patients.

The standard DNA of NA12878 (Charles River Laboratories, Coriell-NA12878) was used to establish the rapid TES pipeline. Peripheral venous blood samples from patients and their parents were collected. Genomic DNA was extracted using the QIAamp DNA Blood Mini Kit (Qiagen, Germany) following the manufacturer’s protocol. The quality and quantity of the DNA samples were measured using the NanoDrop 2000 spectrophotometer (Thermo Fisher Scientific, USA) and Qubit Assay (Thermo Fisher Scientific, USA). One hundred nanograms of genomic DNA were used for library construction. The criteria for genetic testing were approved by the ethics committees of Children’s Hospital, Fudan University (2016-235). Pretest counseling was performed by physicians, and appropriate informed consent was obtained from the patient’s parents. The standard DNA samples and critically ill infant samples followed the standard operating procedure (SOP) in the laboratory. The pipeline for TAT was within 24 h (Fig. 1b).

### Rapid exome sequencing

The rapid TES library was derived from multiple PCR reactions that were conducted using the Ion AmpliSeq™ HiFi Mix and Ion AmpliSeq™ Exome Pool kit, in addition to digestion of the primer sequences (Ion AmpliSeq™ Library Kits 2.0), followed by adaptor and barcode ligation (Ion Xpress Barcode Adapters Kit, Life Technologies, USA). The libraries were purified by Ampure (Beckman, USA), quantified using real-time PCR with the IonTaq Assay (Ion Library Quantification Kit, Life Technologies, USA) and diluted to 100 pM. Then, the trio-family libraries with unique barcodes were mixed. The Ion OneTouch™ system (Life Technologies, USA) was used to clonally amplify the pooled barcoded libraries of the Ion Sphere™ particles, and the chips were loaded. The loaded libraries were sequenced on the Ion Torrent S5 XL (Life Technologies, USA) platform. Torrent Suite™ software was used to compare the base calls and to perform the variant calling. The average on-target sequencing depth for rapid TES was 70×. Based on the NA12878 standard sample, the accuracies of variant calling on SNV and InDel in the regions with effective coverage (read depth ≥ 10×) were 99.79% and 95.05%, respectively. These benchmarks are comparable to a previous publication^25^. (Supplementary Tables 5, 6).

Routine exome sequencing of these samples were performed in parallel using standard protocols in a Clinical Laboratory Improvement Amendments (CLIA) compliant sequencing laboratory in Wuxi NextCODE (288 Fute Zhong Road, Waigaoqiao Free Trade Zone Shanghai 200131, China CLIA ID 99D2064856), with the Agilent SureSelect Human All Exon kit (Agilent Technologies Inc., USA) and Illumina TruSeq Rapid PE Cluster and SBS kit (Illumina, USA). Sequencing was performed using Illumina HiSeq (Illumina, USA).

### Data analysis and molecular diagnoses

Data analysis of the 24-hour rapid TES and regular TES were performed following the bioinformatics data analysis pipeline for next-generation data analysis established in our laboratory (Fudan pipeline)^26^, which is described in detail in the Supplementary Method. On average, after filtering, variants were reduced from 1060 to 433, and the candidate variants were about 25, which needed to be further evaluated by senior genetic reviewers (Supplementary Table 6). Then, the candidate pathogenic variants found in the patients and parents were confirmed by Sanger sequencing on an ABI 3730 Genetic Analyzer (Applied Biosystems, USA). CNV calling was not performed in this study.

### Clinical genetic diagnosis and clinical evaluation

The criteria for variant classification followed the American College of Medical Genetics (ACMG) guidelines^27^. The case was consider diagnosed when pathogenic or likely pathogenic variant(s) were detected in a disease gene, the zygosity of the mutant allele matched the inheritance pattern; additionally, the gene was associated with the patient phenotype^15^. The provisional oral report was provided to ICU clinicians immediately after a diagnosis was made. Genetic experts from the laboratory were invited to join the multidisciplinary consultation teams. The genetic diagnosis propelled the clinical disposition strategy. The quality of the candidate diagnosis variants was also compared between two platforms (see Supplementary Table 8).

### Length of ICU stay and clinical changes in management

Patients staying in the PICU/NICU were treated with standard clinical procedures while the diagnosis was unclear. The daily cost in the ICU was $300–$1000, including clinical care, investigations, clinical treatments, and laboratory examination. The charge for regular TES was $1500 for each patient, including variant validation by Sanger sequencing. The cost of the 24-h rapid TES was covered by the laboratory. The cost of hospitalization was evaluated by the number of days of stay in the ICU.

### Reporting summary

Further information on experimental design is available in the Nature Research Reporting Summary linked to this article.

## Supplementary information

Supplementary Information

Reporting Summary

## Data Availability

The pathogenic variants have been submitted to ClinVar. The accession numbers were listed in Supplementary Table 9. This study is compliant with the ‘Guidance of the Ministry of Science and Technology (MOST) for the Review and Approval of Human Genetic Resources’, which requires formal approval for the export of human genetic material or data from China.
