# Supplementary material for: Clinical utility of 24-h rapid trio-exome sequencing for critically ill infants
Source: NPJ Genom Med. 2020 May 5;5:20. doi: 10.1038/s41525-020-0129-0 (PMC7200743; doi:10.1038/s41525-020-0129-0)
Supplement: Supplementary file 1 — Supplementary Information [file 41525_2020_129_MOESM1_ESM.pdf]

Supplementary data

Clinical utility of 24-hour rapid trio-exome sequencing for critically ill infants

Huijun Wang[1#], Yanyan Qian[1#], Yulan Lu[1], Qian Qin[1], Guoping Lu[2], Guoqiang Cheng[3], Ping Zhang[1], Lin Yang[1], Bingbing Wu[1 *], Wenhao Zhou[1,2 *]

Supplementry Table 1. Clinical information, molecular diagnosis, and changes in management of twenty-three diagnosed patients by rapid TES in NICU/PICU

ID/Sex/ Age (d)	HPO term	Gene: variants	IP/Zygote/Source	Disease[MIM]	TAT (Rapid/ Regular)	Days in ICUs	Clinical managements and changes	Outcome	Benefit from Rapid WES
Case 1/ F/210	Pneumonia; Infantile muscular hypotonia; Seizures; Umbilical hernia; Abnormal myelination; Hypertonia; Severe hearing impairment; Opisthotonus; Neonatal respiratory distress	*GALC:* NM_000153:c.1901T>C, p.L634S; c.869G>A, R290H; c.187_188insAGC, p.G63delinsER	AR/Compound Het/Parents	Krabbe disease [245200]	27H/ 12D	8	Symptomatic treatment, genetic counseling	Alive, in follow up	YES: decrease hospitalization days
Case 2/ F/120	Leukoencephalopathy; Micrognathia; Atria septal defect; Laryngeal cartilage malformation; Pneumonia; Feeding difficulties; Respiratory failure	*KAT6B*: NM_012330: c.4298_4304delinsTC, p.H1433Lfs*29	AD/Het/*De novo*	Ohdo syndrome, SBBYS variant[603736]	24H/ 10D	38	Symptomatic treatment: orthopaedics clinic, ophthalmology clinic, cardiology clinic, and rehabilitation follow-up	Alive, in follow up	NO

Case 3/ F/70	Metabolic acidosis; Dyspnea; Feeding difficulties; Abnormality of the mitochondrion	*EARS2:* NM_001083614: c.193A>G, p.K65E	AR/Hom/Parents	Combined oxidative phosphorylation deficiency 12 [614924]	26H/ 9D	2	Stop medical support	Death	YES: withdraw medical support after received diagnosis
Case 4/ M/11	Neonatal hypoglycemia; Lactic acidosis; Encephalopathy; Seizures; Abnormality of coagulation	*SERAC1:* NM_032861: c.442C>T, p.R148X	AR/Hom/Parents	3-methylglutaconic aciduria with deafness, encephalopathy, and Leigh-like syndrome, MEGDEL[614739]	23H/ 11D	17	Symptomatic treatment: vitamin cocktail and energy mixture treatment. Monitor of blood gas + liver and kidney function + muscle enzyme + hematuria metabolite	Alive, in follow up	YES: early control symptom
Case 5/ M/84	Crohn's disease; Diarrhea; Malnutrition	*IL10RA:* NM_001558: c.537G>A, p.T179T; c.634C>T, p.R212X	AR/Compound Het/Parents	Inflammatory bowel disease 28 [613148]	23H/ 9D	37	Treated with HSCT	Alive, in follow up	YES: early decided to do HSCT
Case 7/ F/28	Lactic acidosis; Diarrhea	*RRM2B:* NM_015713: c. 527_528insG, p.I176Mfs*4; c.128T>A, p.V43D	AR /Compound Het/ Parents	Mitochondrial DNA depletion syndrome 8A[612075]	25H/ 10D	38	Parents finally decided to withdraw medical support	Death	NO
Case 8/ F/28	Seizures; Ventricular septal defect; Pulmonary hypertension; Pulmonary hypertension	*STXBP1:* NM_003165: c.326-1G>T;	AD/Het/*De novo*	Epileptic encephalopathy, early infantile, 4[612164]	24H/ 10D	2	Treated with appropriate antiepileptic drugs: seizure onset at first 2 days of life. phenobarbital, valproic acid, and vigabatrin were suggested to treat seizure, use one or in combination. The family	Alive, in follow up	YES: find ways to control symptom

							decided to continue to treat in a local hospital.		
Case 9/ M/74	Seizures	*KCNQ2:* NM_172107: c.587C>T, p.A196V	AD/Het/*De novo*	Epileptic encephalopathy, early infantile, 7 [613720]	23H/ 11D	10	Treated with appropriate antiepileptic drugs: seizure onset at first 5 days of life but there was no remission with phenobarbital (PB). The seizure was controlled after changed sodium valproate syrup (2ml, bid).	Alive, in follow up	YES: early control symptom
Case 10/ M/33	Thrombocytopenia; Respiratory failure; Anemia; Encephalopathy; Seizures; Metabolic acidosis	*PCCA:* NM_000282: c.1845+1G>A; c.446delA, p.N149Tfs*35	AR/Compound Het/Parents	Propionic acidemia[606054]	24H/ 9D	3	Stop medical support	Death	YES: withdraw medical support after received diagnosis
Case 11/ F/6	Hyperkalemia; Neonatal asphyxia; Abnormality of fatty-acid metabolism; Ventricular fibrillation; Hyperammonemia; Abnormality of carnitine metabolism	*CPT2:* NM_000098: c.764A>G, p.D255G; c.1033G>A, p.G345R	AR/Compound Het/Parents	CPT II deficiency, lethal neonatal [608836]	24H/ 9D	3	Died due to critical illness after received rapid WES result when staying in ICU (ventricular fibrillation, multiple organ dysfunction, upper gastrointestinal bleeding)	Death	NO
Case 12/ F/80	Neonatal asphyxia; Hydrops fetalis; Appendicular hypotonia; Edema; Metabolic acidosis; Pleural effusion;	*FOXC2:* NM_005251: c.361C>T, p.R121C	AD/Het/*De novo*	Lymphedema-distichiasis syndrome, [153400]	24H/ 10D	1	Died due to critical illness before the rapid WES result coming when staying in ICU	Death	NO

	Respiratory distress; Hypothyroidism								
Case 13/ M/81	Muscular hypotonia; Dyspnea; Hypertrophic cardiomyopathy; Pneumonia	*GAA:* NM_000152: c.1935C>A, p.D645E	AR/Hom/Parents	Glycogen storage disease II[232300]	23H/ 10D	8	Plan to treat with enzyme replacement in a local hospital	Alive, lost follow up	YES: acquired precision treatment suggestion. Muscle biopsy was avoided.
Case 14/ M/31	Thrombocytopenia; Anemia; Decreased liver function; Immunodeficiency	*CYBB:* NM_000397: c.1139G>A, p.W380X	XLR/Hemi/De n*ovo*	Granulomatous disease, chronic, X-linked [306400]	24H/ 9D	8	BCG vaccination has performed because of normally at birth. Died of severe infection, septicemia, and MODS, no HSCT	Death	NO
Case 15/ M/6	Appendicular hypotonia; Respiratory failure; Hypoglycemia; Hyperammonemia	*SLC25A20:* NM_000387: c.199-10T>G; c.270delC, p.F91Lfs*38	AR/Compound Het/Parents	Carnitine-acylcarnitine translocase deficiency: [212138]	25H/ 9D	3	Because of apnea, patient was transferred from NICU to PICU, treated with CRRT (continuous renal replacement therapy), and L-carnitine iv.100-200mg/kg.d. Parents decided to withdraw medical support in day 3	Death	YES: withdraw medical support after received diagnosis
Case 16/ F/79	Cardiomyopathy; Immunodeficiency; Hepatosplenomegaly; Diarrhea	*IL7R:* NM_002185: c.221+1G>A	AR/Hom/Parents	Severe combined immunodeficiency, T-cell negative, B-cell/natural killer cell-positive type) [608971]	24H/ 12D	12	Treated with HSCT	Alive	YES:early suggestion for HSCT

Case 17/ M/55	Respiratory failure; Pneumonia; Seizures; Atrial septal defect, Status epilepticus EEG abnormality	*SCN2A:* NM_021007: c.4886G>A, p.R1629H	AD/Het/*De novo*	Epileptic encephalopathy, early infantile, 11 [613721]	24H/ 11D	8	Treated with appropriate antiepileptic drugs: Phenobarbital + levetiracetam + topote combined, convulsions decreased	Alive, in follow up	YES: early control Symptom
Case 18/ M/13	Decreased liver function; Neonatal hypotonia; Pneumonia;	*PEX1:* NM_000466: c.1483+1G>A; c.1725dupG, p.R577Tfs*15	AR/Compound Het/Parents	Peroxisome biogenesis disorder 1A:[214100]	26H/ 11D	3	Withdraw medical support in day 3. Parents inquired prenatal diagnosis and plan to do IVF	Death	YES: withdraw medical support after received diagnosis
Case 20/ M/29	Cryptorchidism; Hypospadias; Highly arched eyebrow; Feeding difficulties; Micrognathia; Microcephaly; Hypertrichosis	*NIPBL:* NM_133433: c.1660C>T, p.Q554X	AD/Het/*De novo*	Cornelia de Lange syndrome 1 [122470]	24H/ 9D	7	Symptomatic treatment	Alive, in follow up	NO
Case 25/ M/30	Osteopetrosis; Anemia; Thrombocytopenia; Hypocalcemia; Hyperphosphatemia; Premature birth; Pulmonary hypertension	*TCIRG1:* NM_006019: c.1114C>T, p.Q372X; c.2008C>T, p.R670X	AR/Compound Het/Parents	Osteopetrosis, autosomal recessive 1[259700]	24H/ 9D	55	HSCT: to be transplanted from brother. However, family decides to sign the abandonment treatment.	Alive	YES: early decided to do HSCT
Case 28/ M/74	Pneumonia; Ventricular septal defect; Hypoplastic heart; Micrognathia; Decreased liver function	*COG6:* NM_020751: c.511C>T, p.R171X; c.540G>A; p.E180E	AR/Compound Het/Maternal, *De novo*	Congenital disorder of glycosylation, type III[614576]	27H/ 10D	72	IVF baby, died in ICU	Death	NO
Case 31/ M/22	Abnormality of the cranial nerves; Abnormality of the	*CHD7:* NM_017780: c.5405-17G>A	AD/Het/*De novo*	CHARGE syndrome[214800]	23H/ 9D	21	Family withdraw because of feeding problem, malformation	Death	NO

	pinna; Abnormal heart morphology; Fever						and severe infection		
Case 32/ M/59	Immunodeficiency	*IFNGR1:* NM_000416: c.295T>C, p.W99R; c.476delT, p.V159Afs*18	AR/ Compound Het/Parents	Immunodeficiency 27A, mycobacteriosis, AR [209950]	23H/ 9D	61	HSCT was performed after two months, but failed because of MODS	Death	YES: early decided to do HSCT
Case 33/ M/87	Pneumonia; Interstitial pulmonary disease; Respiratory failure; Cerebral hypoplasia; Eosinophilia	*CD40LG:* NM_000074: c.359delC, p.P120Lfs*8	XLR/Hemi/Maternal	Immunodeficiency, X-linked, with hyper-IgM: [308230]	25H/ 10D	30	Treated with HSCT	Alive, transplantation status, in follow up	YES: early decided to do HSCT

M mal, *F* female, *D (d)* day, *H* hour, *AR* autosome recessive, *AD* autosome dominant, *BCG* Bacille Calmette-Guerin vaccine, *ER* emergency room, *HSCT* hematopoietic stem cell transplantation, *IVIG* Intravenous immunoglobulin therapy, *MODS* multiple organ dysfunction syndrome, *IP* Inheritance pattern, *XLR* X link recessive.

Supplementary Table 2. Clinical information of false negative of rapid TES of the patients

**ID/Sex / Age (d)**	**HPO term**	**Gene: variants**	**IP/Zygote/Source**	**Disease[MIM]**	**TAT (Rapid/ Regular)**	**Days in ICUs**	**Clinical managements and changes**	**Rapid WES made difference**	**Outcome**
Case 6/ M/51	Respiratory failure; Hyperhomocystinemia; Meningitis; Thrombocytopenia;	*MTHFR:* NM_005957: c.1267dupG; p.E423Gfs*6	AR/Hom/Parents	Homocystinuria due to MTHFR deficiency, [236250]	25H， FN* /21D	31	Betaine, folic acid, cobalamin treatment	NO	Alive, in follow up
Case 19/ M/2	Neonatal asphyxia; Neonatal hypotonia; Respiratory failure;	*MTM1:* NM_000252: c.1446_1447delTG;p.C482*	XLR Hemi Maternal	Myotubular myopathy, X-linked, [310400]	25H FN*/ 25D	9	Palliative therapy	NO	Alive, in follow up

M male, *D (d)* day, *H* hour, *AR* autosome recessive, *XLR* X link recessive, *FN** false negative

Supplementary Table 3. Clinical information and outcome of 8 negative cases in ICU

ID/Sex / Age (d)	HPO ID: Term	Clinical procedures	Outcome	Days in ICUs	TAT (Rapid/ Regular)
Case 21/ F/150	Agammaglobulinemia; Sepsis; Immunodeficiency; Febrile seizures; Atria septal defect; Hepatosplenomegaly; Meningiti; Pneumonia;Respiratory failure; Enterocolitis; Malnutrition	Death in follow up	Death	26	27H/ 10D
Case 22/ M/41	Abnormality of acid-base homeostasis; Respiratory failure; Acute kidney injury; Hypoglycemia;Abnormality of coagulation;Acute hepatic failure	Died in ICU	Death	1	24H/ 10D
Case 23/ F/26	Muscular hypotonia; Pneumonia;Large for gestational age; Abnormality of the ventricular septum	Died in ICU	Death	7	25H/ 10D
Case 24/ M/82	Atelectasis; Encephalopathy; Hypoplasia of the corpus callosum;Seizures; Status epilepticus	Anti-epilepsy	Alive	24	24H/ 9D
Case 26/ M/26	Cryptorchidism; Myopathy;Encephalopathy;Neonatal asphyxia; Pulmonary hypoplasia	Symptomatic treatment	Alive	31	24H/ 10D
Case 27/ M/60	Pneumonia; Respiratory failure;Hyperammonemia	Palliative therapy	Alive	47	22H/ 9D
Case 29/ M/41	Thrombocytopenia; Respiratory failure; Anemia; Encephalopathy; Immunodeficiency; Pulmonary hypertension; Cholestasis; HP:0100806: Sepsis; Ventricular septal defect; Pulmonary hypertension	Death	Death	43	24H/ 11D
Case 30/ F/58	Immunodeficiency; Neonatal respiratory distress; Neonatal hypoproteinemia; Patent foramen ovale; Enterocolitis; Anemia	Palliative therapy	Alive	52	24H/ 9D

Supplementary Table 4. The diagnostic rate by Rapid Trio-WES in the group of the patients with special phenotype

HPO Term	HPO ID	Diagnostic rate with the HPO term (%)
Abnormality of the nervous system	HP:0000707	14/24 (58.3)
Abnormality of the respiratory system	HP:0002086	12/21(57.1)
Abnormality of metabolism/homeostasis	HP:0001939	9/17(52.9)
Abnormality of the immune system	HP:0002715	8/16(50)
Abnormality of the digestive system	HP:0025031	10/16(62.5)
Abnormality of the cardiovascular system	HP:0001626	10/15(66.7)
Abnormality of blood and blood-forming tissues	HP:0001871	5/12(41.7)
Abnormality of the musculature	HP:0003011	4/8(50)
Abnormality of prenatal development or birth	HP:0001197	3/6(50)
Abnormality of the genitourinary system	HP:0000119	2/5(40)
Abnormality of the integument	HP:0001574	4/5(80)

Abnormality of the skeletal system	HP:0000924	3/4(75)
Abnormality of the ear	HP:0000598	2/3(66.7)
Abnormality of cennective tissue	HP:0003549	2/2(100)
Abnormality of head or neck	HP:0000152	2/2(100)
Abnormality of the eye	HP:0000478	1/2(50)
Growth abnormality	HP:0001507	0/2(0)

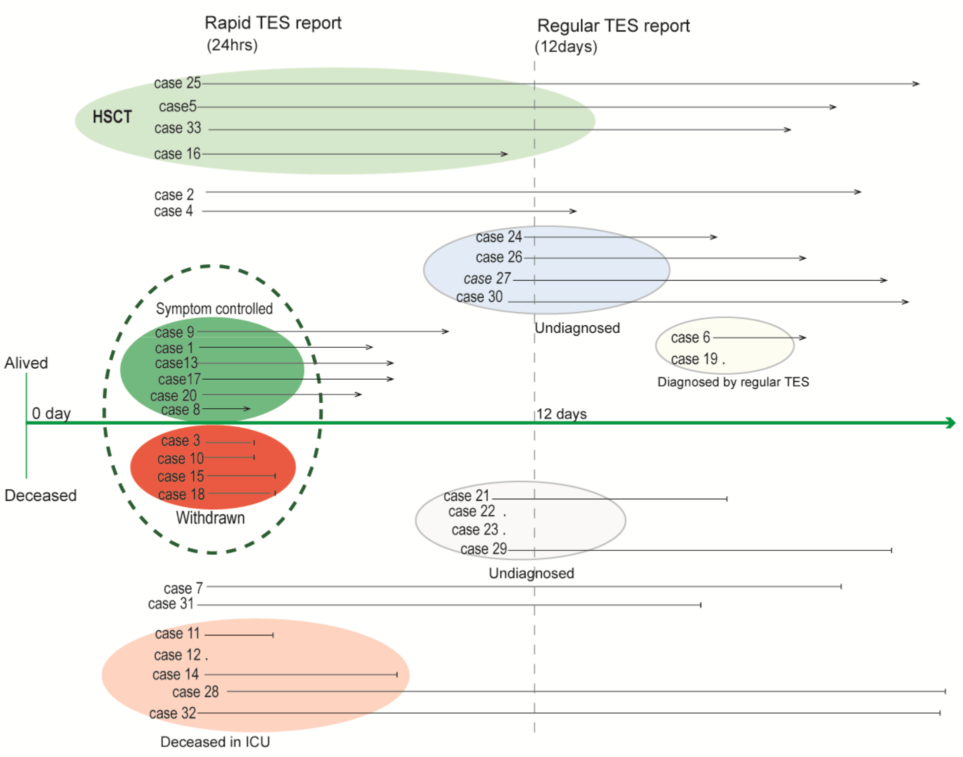

Supplementary Figure 1. Test turnaround time, hospitalization and outcomes of enrolled patients. The horizontal green bold line with arrow indicates the timeline. The filament line with arrow indicates case in follow-up, the filament line with the truncated symbol indicates the case has died. The circle represents a group of cases. The green oval covers six cases with symptoms controlled and discharged before receiving regular TES test results. The red oval represents four cases who were withdrawn from medical support after the rapid TES diagnosis. The hospital stay was saved in case these ten cases (oval in dotted line) after received diagnoses.

Supplemental Method:

1. NA12878 standard DNA sequencing

The variants of NA12878 generated from different platforms were compared against a gold standard truth dataset developed by Genome in a Bottle Consortium (GIAB). The variants of GIAB was download from ftp://ftp-trace.ncbi.nih.gov/giab/ftp/release/NA12878_HG001/latest/GRCh37/HG001_GRCh37_GIAB_highconf_CG-IllFB-IllGATKHC-Ion-10X-SOLID_CHROM1-X_v.3.3.2_highconf_PGandRTGphasetransfer.vcf.gz (version 3.3.2). The platforms contained S5XL of IonAmpliseq Exome and Illumina platform of SureSelect Exome. Read depth coverages of both platfroms were comparatively analyzed.

We applied the tool RTG vcfeval (version 3.9, [Joint Variant and De Novo Mutation Identification on Pedigrees from High-Throughput Sequencing Data. Journal of Computational Biology. June 2014, 21(6): 405-419. doi:10.1089/cmb.2014.0029.]) with lenient haploid comparison (--squash-ploidy) for the comparison, with no other specific parameters.

We tested the NA12878 sample on the Ion Proton platform using the AmpliSeq Exome Kit. Detected variants were compared with the GIAB golden standard. The comparison region (Region A) was first defined as the overlap region of the GIAB high confidence region, the designed capture region of the AmpliSeq Exome, the CDS region of the target gene, the Ion Proton high confidence region (depth ⩾ 10) and the common variants of exome datasets from GNOMAD (AF) ⩾ 1% and AF ⩽ 99%, https://gnomad.broadinstitute.org). SNVs and indels from the gnomAD database were separately used as reference to calculate sensitivity, specificity and accuaracy as following.

Sensitivity = Ture Positive / (Ture Positive+False Negative)

Specificity = Ture Negative / (Ture Negative + False Positive) c

Accuracy = (Ture Positive + Ture Negative) / (Ture Positive+False Negative + Ture Negative + False Positive)

2.The bioinformatics analysis pipeline:

Torrent Suite™ software was used to form the base calls, perform nucleotide sequence alignments, generate FASTQ files and calculate average coverage. The sensitivity and precision of NA12878 in these two platforms were evaluated in the comparison regions as above described. Coverage comparison between two platforms was uniformly computed under the cutoff of mapping quality 5 through bedtools [https://doi.org/10.1093/bioinformatics/btq033]. The Variant Call Format (VCF) of Ion Torrent S5 XL

platform and Illumina were generated by Torrent Suite™ Software and GATK, respectively. The VCF files of Ion Torrent S5 XL platform were also annotated and filtered by Fudan pipeline v2(1--), which annotated variants with NCBI dbSNP, known disease-causing mutations from HGMD and ClinVar, and minor allele frequencies from multiple population datasets (including gnomAD, ExAC, and a local database with over 10k NGS pediatric patients). In addition, the risk of SNVs or indels was predicted by the SIFT (http://sift.jcvi.org/), Polyphen 2 (http://genetics.bwh.harvard.edu/pph2/) and MutationTaster (http://www.mutationtaster.org/) software. The inheritance pattern was scored according to the conformation to help determine pathogenic mutation. The pipeline also extracted Human Phenotype Ontology (HPO) terms from patients' electronic medical record (EMR) through UMLS, as described in Fudan pipeline v2-- (2). After the filtering, the average variants were reduced from 1,060 to 433, and the prioritized variants were 25 on average. The senior genetic reviewers then reviewed the prioritized variants and issued the diagnostic report.

Supplemental Result

The quality of the standard DNA sequencing by the rapid project

We used the standard NA12878 to evaluate the rapid whole exome sequencing result. The library of NA12878 was sequenced in one 540 chipand14.8 gigabase raw data was obtained. Among them, 14.18 Gb could be mapped and 11.74 Gb's quality was more than Q20. The mean depth of the standard was 217.1×.

To match the trio-family sample data, we randomly down-sampled the data of NA12878 to average 70× with 3 replicates and compared the down-sampled data with GIAB golden standard. The average sensitivity, specificity and accuracy is shown in Supplementary Table 1 and Table 2.

To evaluate the Ampliseq Exome sequencing on S5XL of genetic diagnosis in our center, we compared our variant calling results (vcf format) of the NA12878 with the gold standard variants from GIAB study (3, 4). The result of our rapid exome sequencing platform was similar as the reported(5), and the IonTorrent sequencing platform showed much better in SNVs calling(6).

Supplementary Table 5. The results of standard NA12878 detected by different whole exome sequencing platform in coding region of effective coverage region

Comparison Region	Variant Type	Sensitivity	Specificity	Accuracy
Region A	SNV	91.99%	99.998%	99.79%
	INDEL	90.74%	95.16%	95.05%

Supplementaryl Table 6. The results of standard NA12878 detected by different whole exome sequencing platform

GNOMAD_SNV: 65840	GIAB Positive	GIAB Negative
Ion Torrent Called	1584	1
Ion Torrent Not Called	138	64117

GNOMAD_INDEL: 761	GIAB Positive	GIAB Negative
Ion Torrent Called	16	36
Ion Torrent Not Called	2	707

Supplementary Table 7. The number of the variants generated in the rapid TES platform and in regular platform

case number	before filter platform FP			after filter platform FP			Routine platform		
	total	Essential	Candidate	total	Essential	Candidate	total	Essential	Candidate
Case 1	39330	1502	79	39330	810	76	47722	373	45
Case 2	36785	1046	28	36785	416	22	47466	365	26
Case 3	34943	926	24	34943	316	19	48298	574	41
Case 4	38556	1546	74	38556	833	64	47331	361	35
Case 5	34741	896	32	34741	311	21	47015	512	50
Case 6	33566	1350	92	33566	953	43	46566	317	25
Case 7	34769	1580	49	34769	284	10	46468	331	31
Case 8	34116	821	13	34116	524	9	46811	482	28
Case 9	37330	960	16	37330	333	14	46664	362	29
Case 10	35042	853	18	35042	283	16	46732	382	34
Case 11	37408	1002	17	37408	343	14	46543	381	38
Case 12	33491	797	8	33491	230	8	47655	319	37
Case 13	37090	1048	20	37090	397	20	46707	360	35
Case 14	38927	1444	76	38927	784	73	47516	358	48
Case 15	32868	864	19	32868	278	12	47339	355	27
Case 16	37039	996	13	37039	340	8	47131	381	21
Case 17	34548	879	27	34548	299	20	46968	354	43
Case 18	29515	755	20	29515	271	18	46385	364	38
Case 19	34620	1251	71	34620	806	71	46078	596	49
Case 20	34144	861	13	34144	253	11	46787	333	27
Case 21	28985	891	25	28985	367	25	47044	382	29
Case 22	32945	1006	32	32945	431	25	33859	462	42
Case 23	37877	1334	50	37877	761	49	47195	379	31
Case 24	36939	1012	24	36939	381	23	46951	380	39
Case 25	37726	976	22	37726	340	20	46869	355	48
Case 26	37150	903	14	37150	294	10	46017	351	30
Case 27	37572	934	14	37572	322	14	47153	289	36
Case 28	37804	1048	33	37804	389	22	47273	325	40
Case 29	37361	949	18	37361	324	13	44354	330	37
Case 30	37751	896	6	37751	295	5	47185	351	28
Case 31	36943	1092	26	36943	462	24	47319	684	29
Case 32	51292	1270	25	51292	565	24	47481	363	31
Case 33	39004	1260	59	39004	759	55	45996	302	39
**Average**	**36308**	**1059**	**32**	**36308**	**447**	**26**	**46511**	**388**	**35**

VCFs were annotated and filtered by two different criteria automatically: the Essential filtering and the Candidate filtering. These two criteria share the same annotation process but differ in filtering parameters or conditions. The screening conditions of Candidate are more demanding than Essential filtering.

For Essential filtering, the following parameters were applied:

1. Variants should be located within capture region;
2. Allele frequency of the variant should be less than 1% in public database and less than 2% in local database;
3. Variants should be recorded in ClinVar or HGMD, or the influence on amino acid should be either missense or LOF;
4. Variants should be either homozygous (hemi), or compound heterozygous in gene, or heterozygous in disease-associated gene.

Candidate filtering were further applied on Essential filtering result with following parameters:

1. Generally, the allele frequency of the variant should be less than 1% in local database, or the variant has been reported in other patients;
2. The variant were reported as DM or DM? in HGMD, or Pathogenic/Likely-pathogenic in ClinVar, or the variant is LOF, or at least one of three protein damage predictions yielded “damage” (SIFT, Polyphen2, and MutationTaster);
3. The variant should be heterozygous in AD genes, or homozygous/compound heterozygous in AR genes.
4. Apply more strict allele frequency filtration on variants in AD genes: for reported or known pathogenic variants, the allele frequency should be less than 0.5%; for variants with no pathology report, the allele frequency should be less than 0.2%.
5. Variants in AD genes should not be seen in control healthy group and variants on chromosome X should not be seen in control healthy fathers.

Supplementary Table 8. The quality of the data through rapid TES project and regular pipeline

Sample ID	Gender	S5 XL platform					Illumina X-TEN platform		
		Total yeild (Gb)	Mapping data (Gb)	>Q20 (Gb)	Coverage	Pathogenetic variants depth	Mapping data (Gb)	Coverage	Pathogenetic variants depth
Case 1	female		4.84	4.13	75.99	[55:40:95];[22:21:43];[36:41:77]	8.70	108.58	[35:46:81];[44:51:95];[9:7:16]
Case 1F	male	14.4	4.51	3.82	70.14	[60:41:101];[17:28:45];[no_variant]	7.76	88.67	[24:58:82];[36:33:70];[no_variant]
Case 1M	female		4.45	3.82	69.73	[no_variant];[no_variant];[31:17:48]	6.82	77.61	[no_variant];[no_variant];[3:6:9]
Case 2	female		3.14	2.68	48.46	[36:31:67]	13.02	170.27	[81:51:132]
Case 2F	male	11.1	3.72	3.16	57.46	[no_variant]	14.31	183.72	[no_variant]
Case 2M	female		4.01	3.41	62.08	[no_variant]	11.89	151.26	[no_variant]
Case 3	female		6.23	5.28	93.66	[0:155:155]	4.65	246.75	[1:261:262]
Case 3F	male	18.1	5.54	4.7	85.86	[75:40:115]	4.23	236.07	[104:107:211]
Case 3M	female		6.04	5.16	94.19	[76:50:126]	4.27	242.02	[82:57:139]
Case 4	male		4.71	4.02	73.99	[0:78:78]	11.27	142.49	[6:100:106]
Case 4F	male	13.8	4.18	3.58	65.32	[24:26:50]	11.34	142.22	[57:41:98]
Case 4M	female		4.32	3.71	67.6	[34:27:61]	10.46	130.94	[21:40:61]
Case 5	male		5.04	4.22	78.71	[16:28:44];[71:50:121]	11.21	136.79	[35:27:62];[15:22:37]
Case 5F	male	14	4.37	3.66	68.12	[no_variant];[54:59:113]	12.00	147.09	[no_variant];[10:17:27]
Case 5M	female		4.29	3.53	67.44	[21:20:41]; [no_variant]	10.25	122.94	[28:31:59]; [no_variant]
Case 6	male		5.46	4.64	86.39	Low coverage (3 reads)	9.93	123.91	[0:118:127]
Case 6F	male	16.8	5.73	4.88	90.63	Low coverage (2 reads)	11.07	141.21	[44:91:139]
Case 6M	female		4.86	4.17	76.22	Low coverage (2 reads)	10.96	138.71	[65:75:147]
Case 7	female		4.9	4.1	75.99	[36:53:89];[12:5:17]	7.51	97.66	[63:57:120];[37:53:90]
Case 7F	male	13.1	4.31	3.58	66.83	[no_variant];[5:7:12]	7.78	101.7	[no_variant];[45,40:85]
Case 7M	female		3.62	3.03	56.17	[28:25:53]; [no_variant]	7.47	98.48	[66,40:106]; [no_variant]

Case 8	female		5.05	4.16	78.95	[8:23:31]	10.61	132.02	[136:70:206]
Case 8F	male	15.5	4.68	3.84	73.2	[no_variant]	10.00	125.09	[no_variant]
Case 8M	female		5.33	4.38	83.49	[no_variant]	10.13	127.99	[no_variant]
Case 9	male		3.8	3.21	59.94	[39:23:62]	9.37	113.01	[103:92:195]
Case 9F	male	12.8	4.13	3.51	64.78	[no_variant]	10.58	129.64	[no_variant]
Case 9M	female		4.55	3.85	71.02	[no_variant]	10.20	124.99	[no_variant]
Case 10	male		5.31	4.2	82.32	[41:37:78];[26,21:47]	10.84	131.02	[26:55:81];[21,18:40]
Case 10F	male	14.8	4.66	3.69	72.4	[41:25:66]; [no_variant]	12.58	154.3	[69:45:114]; [no_variant]
Case 10M	female		4.56	3.6	70.72	[no_variant];[27,25:52]	12.59	152.62	[no_variant];[26,32:58]
Case 11	female		4.53	3.95	69.91	[63:56:119];[42:44:86]	10.35	128.24	[56:45:101];[30,26:56]
Case 11F	male	13.8	4.27	3.73	66.36	[42:81:123];[no_variant]	9.36	117.03	[43:40:83]; [no_variant]
Case 11M	female		4.48	3.92	70.01	[no_variant];[34:46:80]	11.03	138.35	[no_variant];[36:40:77]
Case 12	female		4.08	3.35	63.84	[20:17:37]	12.96	155.32	[50:57:108]
Case 12F	male	13.6	4.02	3.32	62.33	[no_variant]	11.18	134.3	[no_variant]
Case 12M	female		5.27	4.32	81.24	[no_variant]	10.96	132.79	[no_variant]
Case 13	male		3.72	3.22	57.83	[0:80:80]	11.00	134.95	[2:103:105]
Case 13F	male	12.03	4.11	3.56	64.31	[50:36:86]	10.72	132.86	[75:57:132]
Case 13M	female		3.89	3.38	60.76	[35:47:82]	9.17	113.82	[52:52:104]
Case 14	male		4.8	4.01	73.93	[0:19:19]	12.85	167.22	[0:47:47]
Case 14F	male	14.6	5.03	4.18	78.83	[no_variant]	12.93	168.68	[no_variant]
Case 14M	female		4.17	3.5	65.52	[no_variant]	12.74	163.2	[no_variant]
Case 15	male		4.06	3.33	61.8	[24:26:50];[23:17:40]	10.51	132.23	[74:63:137];[67:65:132]
Case 15F	male	13.5	4.16	3.44	64.71	[27:34:61]; [no_variant]	11.94	149.56	[77:64:141]; [no_variant]
Case 15M	female		4.82	3.99	75.41	[no_variant];[34:36:70]	11.42	144.16	[no_variant];[49:56:106]
Case 16	female	12.2	4.07	3.46	63.57	[0:32:32]	9.39	116.74	[0:117:117]
Case 16F	male		3.91	3.31	60.91	[13:17:30]	9.91	124.57	[54:54:108]

Case 16M	female		3.96	3.82	61.72	[30:20:50]	9.92	123.04	[65:66:131]
Case 17	male		4.73	3.97	74.39	[79:78:157]	10.93	135.64	[91:118:209]
Case 17F	male	13.8	4.24	3.53	66.3	[no_variant]	11.47	143.99	[no_variant]
Case 17M	female		4.17	4.32	64.95	[no_variant]	12.41	158.55	[no_variant]
Case 18	male		3.11	2.67	49.97	[19:19:38];[22,8:30]	10.54	139.62	[22:35:57];[80:77:167]
Case 18F	male	11.9	3.71	3.18	57.47	[23:32:55]; [no_variant]	9.46	122.09	[35:19:54]; [no_variant]
Case 18M	female		4.61	4.06	70.88	[no_variant];[24:18:42]	10.31	134.58	[no_variant];[70:88:168]
Case 19	male		5.68	4.64	86.93	low quality (11 reads)	10.12	131.35	[5:131:136]
Case 19F	male	16.7	4.92	4.02	75.51	low quality (15 reads)	9.87	125.86	[129,140:269]
Case 19M	female		5.61	4.6	84.87	low quality (8 reads)	10.34	131.8	[no_variant]
Case 20	male		5.18	4.38	79.82	[37,38:75]	8.71	105.91	[47:20:67]
Case 20F	male	12	3.05	2.6	49.38	[no_variant]	9.83	120.1	[no_variant]
Case 20M	female		3.17	2.68	49.01	[no_variant]	9.75	118.68	[no_variant]
Case 21	female		5.67	4.83	89.11	negative	10.23	127.45	negative
Case 21F	male	16.6	4.43	3.74	69.3	/	10.21	126.09	/
Case 21M	female		5.79	4.95	91.62	/	8.27	102.87	/
Case 22	male		5.38	4.55	84.73	negative	10.14	126.31	negative
Case 22F	male	13.6	4.41	3.73	69.19	/	9.98	121.48	/
Case 22M	female		3.41	2.87	54.16	/	11.52	130.35	/
Case 23	female		5.24	4.38	81.26	negative	11.86	141.21	negative
Case 23F	male	15.1	5.08	4.27	79.16	/	11.70	139.51	/
Case 23M	female		4.21	3.56	66.09	/	13.05	156.95	/
Case 24	male		5.01	4.18	78.97	negative	12.23	147.68	negative
Case 24F	male	13.1	4.39	3.66	68.44	/	11.73	141.2	/
Case 24M	female		3.14	2.61	48.86	/	12.36	149.37	/
Case 25	male	13.9	4.53	3.88	70.03	[86:91:177];[9:11:20]	11.12	136.77	[7:5:12];[39:35:74]

Case 25F	male		4.41	3.79	68.83	[no_variant];[9:10:19]	11.50	142.32	[no_variant];[55:39:95]
Case 25M	female		4.61	3.97	72.65	[62:70:132]; [no_variant]	11.38	140.51	/;/
Case 26	male		3.87	3.28	60.04	negative	10.32	125.57	negative
Case 26F	male	12.97	4.3	3.61	65.95	/	9.68	120.16	/
Case 26M	female		4.52	3.82	68.93	/	10.32	130.89	/
Case 27	male		4.15	3.28	63.78	negative	8.6	90.8	negative
Case 27F	male	10.8	3.25	2.57	48.98	/	8.69	90.43	/
Case 27M	female		3.13	2.49	45.55	/	9.29	97.34	/
Case 28	male		5.21	4.5	81.28	[4:7:11];[7:4:11]	10.56	132.03	[38:56:94];[50:29:79]
Case 28F	male	12.4	4.33	3.74	67.19	Low coverage; [no_variant]	10.69	132.98	[no_variant];[54:47:101]
Case 28M	female		2.51	2.08	38.94	[0:5:5]; [no_variant]	10.82	133.36	[71:60:132]; [no_variant]
Case 29	male		4.99	4.16	75.84	negative	9.66	118.69	negative
Case 29F	male	13.01	4	3.33	61.64	/	10.01	123.79	/
Case 29M	female		3.51	2.95	54.47	/	10.78	133.56	/
Case 30	female		4.56	3.73	72.29	negative	11.33	138.67	negative
Case 30F	male	13.8	3.85	3.18	61.7	/	11.88	147.03	/
Case 30M	female		4.93	4.08	78.67	/	12.17	149.92	/
Case 31	male		4.41	3.74	67.86	[60:65:125]	4.47	247.33	[117:89:206]
Case 31F	female	12.9	3.99	3.37	61.71	[no_variant]	4.51	252.04	[no_variant]
Case 31M	male		4.25	3.63	66.43	[no_variant]	4.47	252.02	[no_variant]
Case 32	male		4.75	4.06	72.18	[1,4:5];[6,4:10]	12.44	153.27	[104:95:199];[61:41:102]
Case 32F	female	11.9	3.8	3.26	57.42	[4,3:7]; [no_variant]	11.98	137.94	[124:90:215]; [no_variant]
Case 32M	male		2.87	2.47	44.59	[no_variant];[4,8:12]	12.03	141.46	[no_variant];[53:42:96]
Case 33	male		5.3	4.45	91.98	[2:17:19]	8.15	83.69	[0:61:61]
Case 33F	male	14.8	4.75	4	73.56	/[no_variant]	10.82	109.5	[no_variant]
Case 33M	female		4.08	3.46	63.86	[11:8:19]	9.25	94.27	[87:52:139]

Supplementary Table 9. The classification and ClinVar accession number of the variants

Gene symbol	Reference sequence	HGVS	Clinical significance	Allele origin	ClinVarAccession
GALC	NM_000153.3	c.1901T>C	Benign	paternal	SCV001190543
GALC	NM_000153.3	c.869G>A	Likely pathogenic	paternal	SCV001190544
GALC	NM_000153.3	c.187_188insAGC	Uncertain significance	maternal	SCV001190545
KAT6B	NM_012330.3	c.4298_4304delinsTC	Pathogenic	de novo	SCV001190546
EARS2	NM_001083614.1	c.193A>G	Pathogenic	inherited	SCV001190547
SERAC1	NM_032861.3	c.442C>T	Pathogenic	inherited	SCV001190548
IL10RA	NM_001558.3	c.537G>A	Pathogenic	paternal	SCV001190549
IL10RA	NM_001558.3	c.634C>T	Pathogenic	maternal	SCV001190550
RRM2B	NM_015713.4	c.527_528insG	Pathogenic	paternal	SCV001190551
RRM2B	NM_015713.4	c.128T>A	Likely pathogenic	maternal	SCV001190552
STXBP1	NM_003165.3	c.326-1G>T	Pathogenic	de novo	SCV001190553
KCNQ2	NM_172107.3	c.587C>T	Pathogenic	de novo	SCV001190554
PCCA	NM_000282.3	c.1845+1G>A	Pathogenic	paternal	SCV001190555
PCCA	NM_000282.3	c.446delA	Pathogenic	maternal	SCV001190556
CPT2	NM_000098.2	c.764A>G	Likely pathogenic	paternal	SCV001190557
CPT2	NM_000098.2	c.1033G>A	Likely pathogenic	maternal	SCV001190558
FOXC2	NM_005251.2	c.361C>T	Pathogenic	de novo	SCV001190559
GAA	NM_000152.4	c.1935C>A	Pathogenic	inherited	SCV001190560
CYBB	NM_000397.3	c.1139G>A	Likely pathogenic	de novo	SCV001190561
SLC25A20	NM_000387.5	c.199-10T>G	Pathogenic	paternal	SCV001190562
SLC25A20	NM_000387.5	c.270delC	Pathogenic	maternal	SCV001190563

IL7R	NM_002185.4	c.221+1G>A	Pathogenic	inherited	SCV001190564
SCN2A	NM_021007.2	c.4886G>A	Likely pathogenic	de novo	SCV001190565
PEX1	NM_000466.2	c.1483+1G>A	Pathogenic	paternal	SCV001190566
PEX1	NM_000466.2	c.1727dupG	Pathogenic	maternal	SCV001190567
NIPBL	NM_133433.3	c.1660C>T	Likely pathogenic	de novo	SCV001190568
CHD7	NM_017780.3	c.5405-17G>A	Pathogenic	de novo	SCV001190569
IFNGR1	NM_000416.2	c.295T>C	Likely pathogenic	paternal	SCV001190570
IFNGR1	NM_000416.2	c.476delT	Pathogenic	maternal	SCV001190571
TCIRG1	NM_006019.3	c.1114C>T	Pathogenic	paternal	SCV001190572
TCIRG1	NM_006019.3	c.2008C>T	Pathogenic	maternal	SCV001190573
COG6	NM_020751.2	c.511C>T	Pathogenic	maternal	SCV001190574
COG6	NM_020751.2	c.540G>A	Likely pathogenic	paternal	SCV001190575
CD40LG	NM_000074.2	c.359delC	Pathogenic	maternal	SCV001190576

Reference:

1. Chen X, Wang H, Wu B, et al. One novel 2.43Kb deletion and one single nucleotide mutation of the INSR gene in a Chinese neonate with Rabson-Mendenhall syndrome. Journal of clinical research in pediatric endocrinology 2017.
2. Bodenreider O. The Unified Medical Language System (UMLS): integrating biomedical terminology. Nucleic acids research 2004;32(Database issue):D267-270.
3. Zook JM, Catoe D, McDaniel J, et al. Extensive sequencing of seven human genomes to characterize benchmark reference materials. Scientific data 2016;3:160025.
4. Zook JM, Chapman B, Wang J, et al. Integrating human sequence data sets provides a resource of benchmark SNP and indel genotype calls. Nature biotechnology 2014;32(3):246-251.
5. Samorodnitsky E, Jewell BM, Hagopian R, et al. Evaluation of Hybridization Capture Versus Amplicon-Based Methods for Whole-Exome Sequencing. Human mutation 2015;36(9):903-914.
6. Damiati E, Borsani G, Giacopuzzi E. Amplicon-based semiconductor sequencing of human exomes: performance evaluation and optimization strategies. Human genetics 2016;135(5):499-511.
